# The Storage Conditions of High-Fat Diet Are the Key Factors for Diet-Induced Obesity and Liver Damage

**DOI:** 10.3390/nu14112222

**Published:** 2022-05-26

**Authors:** Chuanyou Yi, Dandan Li, Xiao Guo, Jinhui Wang, Cenxi Liu, Guangxing Lu, Yan Sun, He Huang, Shangyu Hong, Jin Li

**Affiliations:** 1 State Key Laboratory of Genetic Engineering, School of Life Sciences, Human Phenome Institute, and Institute of Metabolism and Integrative Biology, Fudan University, Shanghai 200438, China; 19110700023@fudan.edu.cn (C.Y.); 20210700108@fudan.edu.cn (D.L.); 19210700052@fudan.edu.cn (X.G.); 19210700151@fuan.edu.cn (J.W.); 20210700024@fudan.edu.cn (C.L.); 20110700051@fudan.edu.cn (G.L.); 2 Masonic Medical Research Institute, 2150 Bleecker St., Utica, NY 13501, USA; ysun@mmri.edu

**Keywords:** high-fat diet, storage condition, obesity, liver damage, medium-chain triglyceride, unfolded protein response, NNMT

## Abstract

The diet-induced obesity (DIO) mouse model has been widely used for obesity studies. The effects of storage conditions on the composition of nutrients in high-fat diets (HFDs) and their impact on metabolic homeostasis have not been systemically investigated. In the current study, we tested the effects of HFDs stored under different conditions and found that mice fed a HFD stored in the fridge (HFD^fri^) gained less weight than those fed HFDs stored in the freezer (HFD^fre^). Further analysis revealed that changes in the relative abundance of medium-chain triglyceride (MCT) in the HFD^fri^, which have much lower intestinal absorption rates, contributed to the body weight differences. In contrast, exacerbated liver damage and elevated levels of unfolded protein response (UPR) was observed in the mice fed by HFD^fri^. Depletion of the UPR-regulated gene *Nnmt* alleviated liver damage via the inhibition of the integrated stress response (ISR). Our study, for the first time, provides evidence that HFD storage conditions can have a significant impact on both body weight changes and liver damage in the DIO model.

## 1. Introduction

Obesity has been recognized as an important chronic disease, which leads to various severe complications such as non-alcoholic fatty liver disease (NAFLD), non-alcoholic steatohepatitis (NASH), and cardiovascular diseases [1,2,3,4]. The diet-induced obesity (DIO) mouse model has been widely used for obesity studies [5], and several kinds of special diets, including high-fat diets (HFDs, 65% energy from fat) and high-fat–high-sucrose diets (HFHSDs, 40% energy from fat and 45% from sucrose), were used to establish the DIO model.

Though some investigators have customized their own diets for the establishment of the DIO model, most investigators use standardized diets provided by vendors such as Research Diets, Inc. (New Brunswick, NJ, USA) or *LabDiet* (Quakertown, PA, USA). These vendors also provide other kinds of diets for obesity studies, such as a high-protein–low-carbohydrate diet or a methionine- and choline-deficient (MCD) diet. The employment of various kinds of standardized diets has reduced the experimental variations introduced by the diet itself, which has helped us to better understand the mechanism of obesity and develop specific therapies for its treatment.

In comparison, the impact of the storage conditions on the nutritional components of the HFDs and their impact on metabolic homeostasis have been generally ignored. The storage conditions are obviously an important factor in the food industry. For biomedical experiments, the HFD is stored at low temperatures at all times. Normally, the HFD is stored in dry ice for transportation, and then stored in either a freezer (−20 °C) or a deep freezer (−80 °C) for long-term storage. Once the package is opened, the investigators may store the HFD in either a fridge (4 °C) or a freezer in the animal facility. The decision is normally dependent on the equipment provided by their animal facility. It is intriguing to study whether the storage conditions for a HFD, after the package has been opened, contribute to the phenotypic changes of the DIO model.

Nicotinamide N-methyl transferase (NNMT) is an enzyme that converts nicotinamide (NAM) to N1-methylnicotinamid (MNAM), and is therefore responsible for the regulation of the abundance of NAD+. The important role of NNMT in maintaining metabolic homeostasis has been investigated in various contexts [6,7,8]. Notably, the expression of *Nnmt*can be regulated by the unfolded protein responses (UPR)-related protein kinase R-like endoplasmic reticulum kinase (PERK)-activating transcription factor 4 (ATF4) signaling in the context of either alcohol/palmitate-induced liver damage [9,10], or carbon tetrachloride-induced NASH [11]. In contrast, its effects on dietary-induced liver damage and the relevant mechanisms have not been studied.

Driven by pure curiosity, we tested the effects of HFD stored in different conditions on young male C57 BL6/J mice. Surprisingly, mice fed byHFDs stored in the fridge (HFD^fri^) gained weight significantly slower than those fed HFDs stored in the freezer (HFD^fre^). A mechanistic study revealed that changes in body weight are related to the low absorption efficiency of medium-chain triglycerides (MCT), which are enriched in the HFD^fri^. Interestingly, the conditions of HFD^fri^ led to activated unfolded protein responses (UPR), enhancement of PERK-ATF4-NNMT signaling, and exacerbated liver damage. Hepatocyte-specific knockout (KO) of *Nnmt* inhibited the integrated stress response (ISR) and protected the liver from MCD diet-induced liver damage.

## 2. Materials and Methods

**Animal Experiments:** All animal experiments were performed according to procedures approved by the Fudan University School of Life Science ethical committee. Wild-type C57BL6/J mice were obtained from GemPharmatech. Eight-week-old male mice were fed by HFD^fri^ or HFD^fre^ side-by-side for seven weeks in the animal facility of Fudan University (specific pathogen free level). The *Alb-Cre* mice and *Nnmt ^flox/flox^* mice were ordered from VIEWSOLID BIOTECH. Mice were maintained under a 12 h light/12 h dark cycle at constant temperature (23 °C), with free access to food and water. The HFD was sealed and stored in −80 °C for long-term storage. Once the package was opened, the HFDs were stored in −20 °C or 4 °C for 7 days before use. The HFD was changed every 3 or 4 days for the mice. The HFD used in the current study was ordered from Research Diet (Cat# D12492). The HFD was stored in −80 °C for 4–12 weeks after the shipment arrived. The HFD diet was moved from −80 °C to −20 °C seven days before the experiments started and stored in −20 °C until the end of the experiments. For every experiment, a new batch of HFDs was moved from −80 °C to −20 °C freshly. The storage time of the HFD used is consistent between the HFD^fri^ and HFD^fre^. Male *Nnmt* KO mice or control mice were fed on a methionine- and choline-deficient (MCD) diet for 8 weeks.

**Lipid Profile:** Blood samples were collected through the tail vein or eyeball and centrifuged at 4000 rpm for 10 min to collect serum. Serum samples were stored at −80 °C. Commercial kits were used to measure triglyceride (TG), total cholesterol (TC), low-density lipoprotein cholesterol (LDL-c), and high-density lipoprotein cholesterol (HDL-c), according to the manufacturer’s guidelines (Nanjing Jiancheng Bioengineering Institute, Nanjing, China).

**Oral Lipid Tolerance Test:** Unless specified, eight-week-old male C57 BL6/J mice without exposure to HFD^fri^ or HFD^fre^ were used for the Oral Lipid Tolerance Test (OLTT experiment). The mice were fasted for 16 h and administrated with sunflower oil (8001-21-6, Sigma; 3.90 g/kg) or medium-chain triglyceride (MCT) (the Nisshin OilliO Group, Ltd.; 4.12 g/kg) via oral gavage. Blood samples were collected via tail vein at 0, 1, 2, 3, 5, and 8 h after the oral gavage, and the serum was acquired by centrifuging the samples at 4000 rpm for 10 min. Commercial kits were used to measure TG and TC concentrations according to the manufacture’s guidelines (Nanjing Jiancheng Bioengineering Institute).

**Lipid and Metabolite Extraction:** The extraction method was modified from a published article [12], and is described briefly here. Serum was collected by centrifuging at 4000 rpm for 10 min at 4 °C from either mice fed HFD^fri^ or HFD^fre^, and 50 µL was transferred to a new glass centrifuge tube. Then, 200 µL HPLC-grade water, 1 mL HPLC-grade methanol, and 5 mL high-performance liquid chromatography (HPLC) methyl tert-butyl ether (MTBE) were vortexed for 1 min. The mixture was then incubated on a rotator for 1 h at room temperature; then, 1.5 mL HPLC-grade water was mixed and vortexed for 1 min. The mixture was separated into two phases after centrifuging at 1000× *g* for 10 min at 4 °C; non-polar lipids and aqueous metabolites were contained in the upper phase and the lower phase, respectively. A SpeedVac was used to dry the non-polar lipids and aqueous metabolites at room temperature. The dried lipids or metabolites were stored at −80 °C until analysis by a liquid chromatograph mass spectrometer (LC-MS).

**Untargeted Lipidomics:** A mixture of 2-propanol:acetonitrile:water (*v:v:v* 30:65:5; 200 µL) was used to reconstitute the nonpolar lipids. A 5 µL reconstituted sample was used for analysis by liquid chromatograph mass spectrometer (LC-MS). The untargeted lipidomics method was modified from a published method [13] by using a C30 column (Acclaim C30, 3 µm, 2.1 × 150 mm). The mass-spec data were acquired using an Orbitrap Exploris 480 (Thermo Fisher Scientific, Shanghai, China), utilizing the polarity-switching approach with data-dependent acquisition (DDA) mode enabled. All lipidomics RAW files were processed on *LipidSearch* 4.0 (Thermo Fisher Scientific) for lipid identification.

**Targeted Metabolomics:** A total of 100 µL acetonitrile:water (*v:v* 50:50) was used to reconstitute the aqueous metabolites. A 5 µL reconstituted sample was used for analysis by LC-MS. The targeted metabolomics method was modified from a published protocol [14] by using an amide hydrophilic interaction liquid chromatography (HILIC) column (XBridge Amide 3.5 µm, 4.6 × 100 mm). The mass data were acquired by a QTRAP 5500+ (AB Sciex) using the polarity-switching approach. The LC-MS/MS peak integration was determined on MultiQuant (AB Sciex), and data were transferred to the metabolomics spreadsheet.

**Metabolomic and lipidomic data analyses:** The MetaboAnalyst 5.0 and LINT-web website was utilized to analyze metabolomic and lipidomics results. In brief, the data was normalized to the median value, log-transformed, and auto-scaled. Then, the relative abundance was calculated based on the processed data. The details of how these tools work are described in the literature [15,16].

**Western Blotting:** Tissues were lysed in 1% NP40 buffer with protease and phosphatase inhibitors. Total protein was determined using a BCA Protein Assay Kit (23225; Thermo Fisher Scientific). Proteins were then separated by 10% SDS-PAGE and transferred to PVDF membranes (Merck Millipore, Beijing, China). The PVDF membranes with proteins were incubated with various specific primary antibodies (Supplementary Table S1) overnight at 4 °C, and then incubated with HRP-conjugated secondary antibodies for 60 min. ECL Reagent (34577, Thermo Fisher Scientific) and a Clinx ChemiScope 6000 (Clinx Science Instruments Co., Ltd., Shanghai, China) were used for visualizing protein bands.

**Histology:** The procedure of immunohistochemistry (IHC) was conducted, and is described briefly as follows: the liver was fixed in 10% formalin for paraffin-embedding, sectioning, and staining. Picrosirius red staining was used to identify the collagen histochemistry with a picro-sirius red reagent (Servicebio, GB1018). The MMP2 staining used an anti-MMP2 rabbit monoclonal antibody (mAb) (Servicebio, GB11130), and the secondary antibodies are HRP-goat antirabbit IgG (H + L) (Servicebio, GB23303). The sections were reacted with DAB kit (DAKO, K5007). Histochemical staining of liver was visualized with a microscope (CIC, XSP-C204) using a 20× objective lens. The quantification of MMP2 staining was performed by initially processing the images with a plug-in software called “IHC Profiler” in ImageJ. The processed MMP2 images and the original PSR images were refined by the “image->adjust->threshold” function and quantified by the ”analyze->measure” function of ImageJ.

**Body Composition:** Body composition was acquired using an Echo MRI (Echo Medical Systems, Houston, TX, USA) with a 3-in-1 Echo MRI Composition Analyzer in order to determine lean mass and fat mass values prior to biochemical experiments.

**Indirect calorimetry:** The energy expenditure was determined by the Promethion Comprehensive Lab Animal Monitoring System (CLAMS, Sable Systems International, NV, USA) housed within a temperature-controlled environmental chamber at Fudan University. A total of 8 male C57Bl/6J mice at 8–10 weeks of age were purchased and fed with HFDs kept under different storage conditions. Before the experiment started, the mice were acclimatized in the CLAMS for 48 h and monitored for 48 h. The data were processed as previously described [17].

**Energy Content Test:** The energy content of diets and feces was measured using a bomb calorimeter (IKA, Guangdong, China) according to the manufacturer’s protocol.

**Glucose and Insulin Tolerance Testing:** For the glucose tolerance test (GTT), mice were fasted for 16 h and received 1 g/kg body weight glucose by i.p. Blood samples were collected and glucose levels were monitored with a portable glucometer at 0, 15, 30, 60, 90, and 120 min time points after i.p. For the insulin tolerance test (ITT), mice were fasted for 5 h and received 0.3 U/kg body weight insulin by i.p. Blood glucose levels were also measured at 0, 15, 30, 60, 90, and 120 min time points after i.p.

**RNA-seq Assay:** Total RNA was extracted from the liver using TRIzol reagent (Thermo Fisher Scientific). Extracted RNA was then reversely transcribed using the PrimeScript™ RT reagent Kit (Takara). RNA-seq libraries for expression analysis were constructed using KAPA RNA HyperPrep Kit KR1350 v1.16, according to the vendor’s protocol, and paired-end 2 × 150 bp reads were sequenced using the Illumina HiSeq platform. The raw data were aligned and quantified by HISAT2 [18]. The raw data was deposited to the Gene Expression Omnibus database at GSE203040. Differentially expressed genes were determined by DESeq2 [19], and *p* values < 0.05 and Log_2_ fold changes > 2 were recognized as the cutoff.

**RT-qPCR:** Total RNA was extracted from tissues with TRIzol (Thermo Fisher Scientific). Extracted RNA (500 ng) was converted into cDNA using the PrimeScript™ RT reagent Kit (Takara). Quantitative RT-PCR (qRT-PCR) was performed with SYBR Green PCR Master Mix (Applied Biosystems, Waltham, MA, USA) in an Applied Biosystems QuantStudio 5. The internal reference genes *36b4* or *Tbp* were served as internal reference genes. The primer sequences are provided in Supplementary Table S2.

**Statistics:** Gene set enrichment analyses (GSEAs) were performed according to their guidelines [20,21]. The Student *t*-test, two-way repeated-measures ANOVA analysis, and a post hoc test of multiple comparison tests where ANOVA found significance were used in this study. The exact *p*-values are provided in Supplementary Table S3.

## 3. Results

### 3.1. Lipid Absorption Efficiency Contributed to Body Weight Differences Induced by HFD Storage Conditions

**Figure 1:**
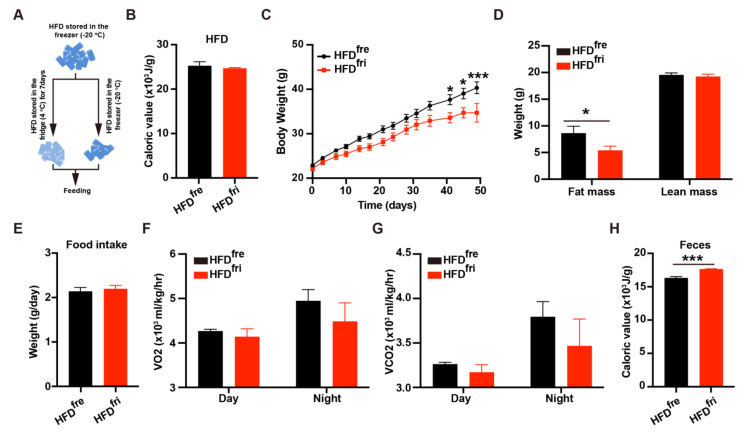
High-fat diets (HFDs) stored in different conditions leads to bodyweight differences. (**A**) The simplified protocol for high-fat diet (HFD) storage. HFDs were divided into two parts. HFDs stored in the freezer (fre) (HFD^fre^) were stored at −20 °C for 7 days, and HFDs stored in the fridge (fri) (HFD^fri^) were stored at 4 °C before the experiments started; (**B**) the bomb calorimeter was used to detect the energy content in HFD^fre^ and HFD^fri^ (HFD^fre^, n = 5; HFD^fri^, n = 6); (**C**–**G**) analysis of metabolic features of the mice. The features include (**C**) body weight (*p* < 0.0001 from two-way ANOVA) (n = 9 for each group), (**D**) body composition (n = 10 for each group), (**E**) food intake (n = 9 for each group), (**F**) average O_2_ consumption rate (VO2, *p* = 0.2766 from two-way ANOVA) (n = 4 for each group), and (**G**) average CO_2_ production rate (VCO2, *p* = 0.2650 from two-way ANOVA) (n = 4 for each group); (**H**) fecal energy content of mice fed by HFDs stored in different conditions (n = 5 for each group). The adjusted *p*-value from multiple comparison tests are indicated as *, *p* < 0.05; ***, *p* < 0.001. The results of the Student *t*-test are provided in Supplementary Table S3.

The young male wild-type C57 BL6/J mice were fed HFDs stored in either a fridge or a freezer (Figure 1A). The detailed storage protocol is described in the Section 2. The energy content in the diet was tested prior to the start of the experiment by bomb calorimetry. No difference in energy content was observed between the two types of HFD (Figure 1B). However, a consistently lower body weight was observed in the mice fed by HFD^fri^ when compared to those fed by HFD^fre^ (Figure 1C). Body composition analysis revealed less fat mass—but not lean mass—in the HFD^fri^ mice (Figure 1D).

To elucidate the reasons for body weight differences, we systemically analyzed the metabolic features of these mice. No difference was observed in food intake (Figure 1E) and water intake (Supplementary Figure S1A). The results of a Comprehensive Lab Animal Monitoring System (CLAMS) analysis indicated the energy expenditure was comparable between mice fed by the two different types of HFD (Figure 1F,G and Supplementary Figure S1B,C). Notably, the mice fed by HFD^fri^ showed an impaired circadian rhythm of energy expenditure, whose mechanism needs to be studied in the future. The physical activities of these mice were similar (Supplementary Figure S1D). The expression of thermogenic genes and proteins in the adipose tissue was not changed by different types of HFD (Supplementary Figure S1E–G). The expression of lipolytic genes and proteins in adipose tissues was also not dramatically changed (Supplementary Figure S1H,I).

In addition to energy expenditure and food intake, body weight can also be regulated by the absorption efficiency of food. Interestingly, more energy content was detected in the feces of mice fed by HFD^fri^ (Figure 1H). Because the majority of energy in HFDs is derived from lipids, we first tested the lipid absorption efficiency in mice fed different types of HFD. No difference was observed in the oral lipid tolerance test (OLTT) with sunflower oil [22] between the two groups of mice with exposure to HFD (Supplementary Figure S1J), indicating the HFD^fri^ did not change the physiological capability of lipid absorption in the mice. Therefore, we hypothesized that the difference in fecal energy content was rather induced by differences in the diet as a result of storage conditions.

### 3.2. The Enrichment of MCT in HFD^fri^ Is Correlated to the Diet-Induced Body Weight Differences

**Figure 2:**
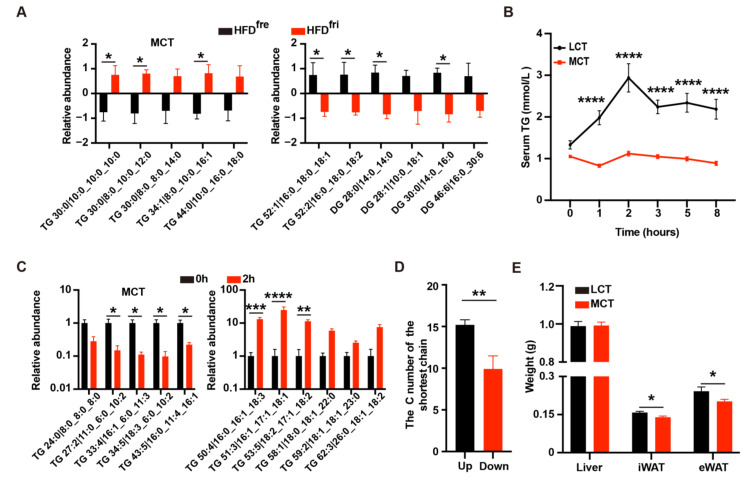
Medium-chain triglycerides (MCT) induced phenotypic changes in the mice fed by high-fat diets (HFD) stored in the fridge (fri) (HFD^fri^). (**A**) Lipidomics analysis of the diet revealed increases of medium-chain triglycerides (MCT) (left panel, *p* < 0.0001 from two-way ANOVA) and decreases of long-chain triglyceride (LCT)/long-chain diglyceride (LCD) (right panel, *p* < 0.0001 from two-way ANOVA) in high-fat diets (HFD) stored in the fridge (fri) (HFD^fri^) (n = 3 for each group); (**B**) the oral lipid tolerance test (OLTT) with MCT-enriched oil or sunflower oil in wild-type mice without exposure to HFDs (*p* < 0.0001 from two-way ANOVA) (n = 10 for each group); (**C**) relative abundance of MCT (left panel, *p* < 0.0001 from two-way ANOVA) and LCT (right panel, *p* < 0.0001 from two-way ANOVA) in the serum of mice in the initial or two-hour time point of OLTT (n = 3 for each group); (**D**) the average length of shortest chain for triglycerides (TG) with high (up, n = 48) or low (down, n = 10) absorptive efficiency; (**E**) the weight of the liver, inguinal white adipose tissue (iWAT), and epididymal white adipose tissue (eWAT) in the 8-week-old male mice fed by MCT-enriched oil or sunflower oil for three weeks (n = 9 for each group). The adjusted *p*-values from multiple comparison tests are indicated as *, *p* < 0.05; **, *p* < 0.01; ***, *p* < 0.001; ****, *p* < 0.0001. The results of the Student *t*-test are provided in Supplementary Table S3.

Untargeted lipidomics was used to analyze the lipid composition of HFD^fri^ and HFD^fre^. Notably, remarkable upregulation of the relative abundance for MCT and downregulation of long-chain triglyceride (LCT)/long-chain diglyceride (LCD) were discovered in HFD^fri^ (Figure 2A, Figures S2A and S4).

To validate whether MCT content differences affected the absorption efficiency, the oral lipid tolerance test (OLTT) was performed in wild-type mice with either MCT-enriched oil or sunflower oil (mixture of MCT and LCT) containing equal amounts of triglycerides (TG). In comparison to sunflower oil, mice receiving MCT-enriched oil via oral gavage presented lower serum concentrations of TG and total cholesterol (TC) in OLTT (Figure 2B and Supplementary Figure S2B). Next, we analyzed the lipid absorption profile for sunflower oil on the serum collected in the initial and two-hour time points of wild-type mice. The serum relative abundance of LCT in the two-hour time point was much higher than MCT in mice (Figure 2C, Supplementary Figure S2C and Table S5). In consistence, the carbon number of upregulated TG in the two-hour time point was higher than the downregulated TG (Figure 2D). These results indicated the LCT has a higher absorption efficiency than MCT.

We then studied the effects of MCT-enriched oil or sunflower oil on the obesity-related phenotypes by treating mice with oil containing equal amounts of energy via oral gavage. After two weeks of treatment, we did not observe dramatic body weight differences (Supplementary Figure S2D). However, consistent with our observations of the mice fed HFD^fri^, the mice fed MCT-enriched oil had lower-weight inguinal white adipose tissue (iWAT) and epididymal white adipose tissue (eWAT) (Figure 2E). Therefore, the changes of lipid absorption efficiency and body composition induced by HFD^fri^ were likely due to the high abundance of MCT in the diet.

### 3.3. HFD^fri^ Led to Exacerbated Liver Damage

**Figure 3:**
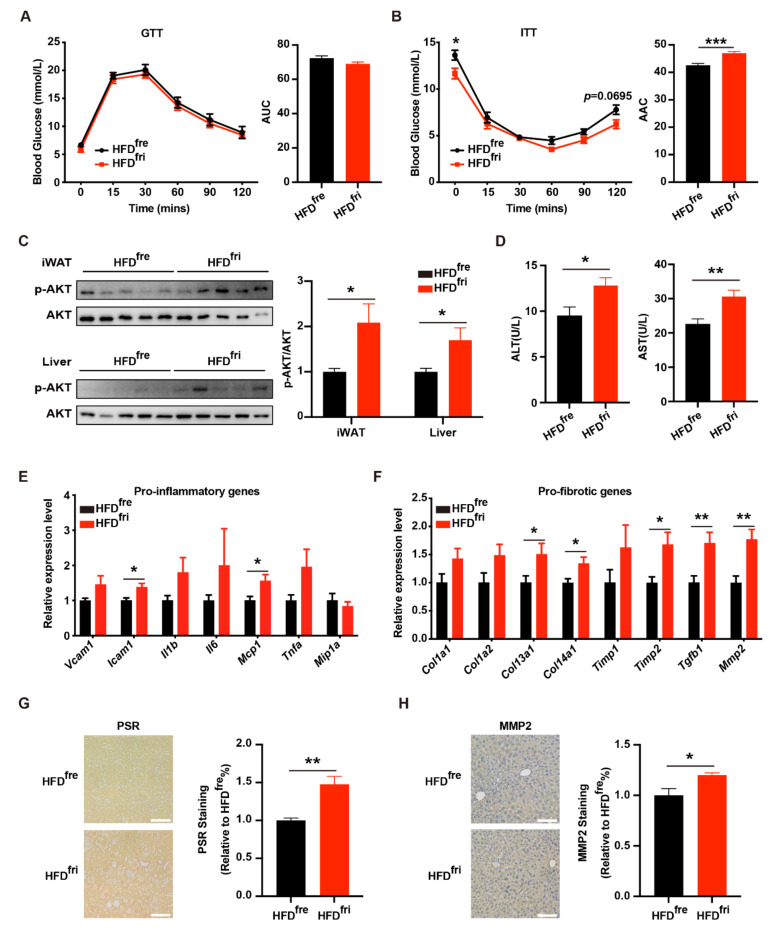
Exacerbated liver damage of the mice fed by high-fat diets (HFDs) stored in the fridge (fri) (HFD^fri^). (**A,B**) Analysis of glucose metabolic conditions with (**A**) glucose tolerance test (GTT) (*p* = 0.1480 from two-way ANOVA) and (**B**) insulin tolerance test (ITT) (*p* < 0.0001 from two-way ANOVA) (for the high-fat diet (HFD) stored in the freezer (fre) (HFD^fre^), n = 8; for the HFD stored in the fridge (fri) (HFD^fri^), n = 7); (**C**) expression of phospho-protein kinase B (*p*-AKT) in the liver, inguinal white adipose tissue (iWAT), and epididymal white adipose tissue (eWAT) in insulin assay of the mice fed by HFD for 7 weeks (n = 5 for each group); (**D**) serum concentration of alanine aminotransaminase (ALT) and aspartate aminotransferase (AST) (HFD^fre^, n = 9; HFD^fri^, n = 7). (**E**) Expression of proinflammatory genes and (**F**) pro-fibrotic genes in the liver of mice fed by HFD (HFD^fre^, n = 8; HFD^fri^, n = 7). (**G,H**) Representative images of Picro Sirius Red (PSR) staining (**G**) and Matrix Metallopeptidase 2 (MMP2) staining (**H**) on liver (n = 6 for each group). Eight-week-old male mice were fed by HFD^fre^ and HFD^fri^ for 7 weeks. Scale bar represents 100 µm. The adjusted *p*-values from multiple comparison tests are indicated as *, *p* < 0.05; **, *p* < 0.01; ***, *p* < 0.001. The results of the Student *t*-test are provided in Supplementary Table S3.

We then tested the effects of different types of HFD on metabolic homeostasis. The mice fed HFD^fri^ presented similar performance on the glucose tolerance test (GTT, Figure 3A) and better performance on the insulin tolerance test (ITT, Figure 3B). The insulin assay also revealed a stronger response to insulin stimulation in the liver and the iWAT of mice fed HFD^fri^ (Figure 3C). However, the serum concentration of alanine aminotransaminase (ALT) and aspartate aminotransferase (AST) was elevated in mice fed HFD^fri^ (Figure 3D). Notably, the inflammation and fibrosis-related genes were upregulated in the livers of these mice (Figure 3E,F). In addition, we observed the elevation of both the Picro Sirius Red (PSR) staining signal (Figure 3G) and Matrix Metallopeptidase 2 (MMP2) staining signal (Figure 3H) in the liver from the mice fed by HFD^fri^. These results suggest that HFD^fri^ induced exacerbated liver damage in comparison to HFD^fre^.

### 3.4. HFD^fri^ Activated UPR-NNMT Signaling in the Liver

**Figure 4:**
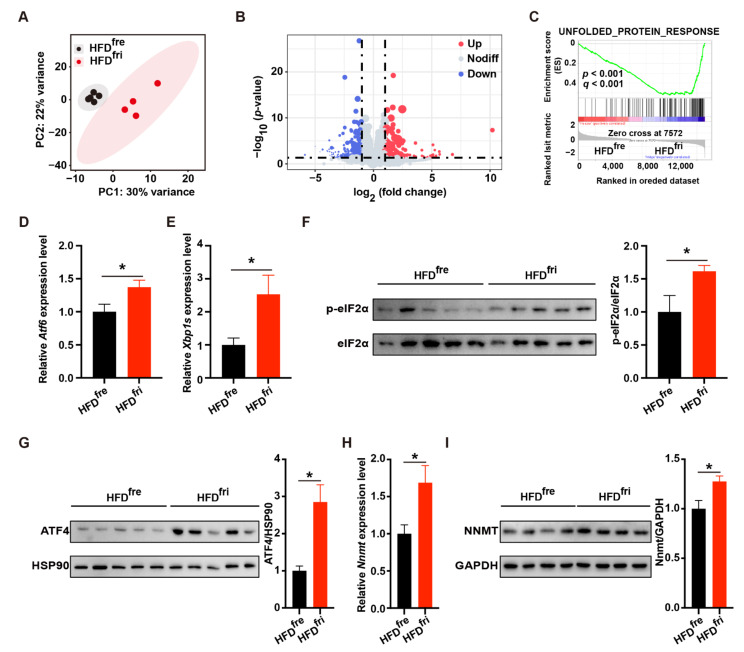
Elevation of unfolded protein response (UPR) in the mice fed by high-fat diets (HFDs) stored in the fridge (fri) (HFD^fri^). (**A**–**C**) RNA-seq analysis of the liver including (**A**) principal component analysis (PCA), (**B**) volcano plot, and (**C**) gene set enrichment analysis (GSEA) results of unfolded protein response (UPR)-related gene set (high-fat diet (HFD) stored in the freezer (fre) (HFD^fr^^e^), n = 5; HFD stored in the fridge (fri) (HFD^fri^), n = 4); (**D**,**E**) relative expression of *activating transcription factor6 (Atf6)* (**D**) and *the spliced form of X-box binding protein 1 (Xbp1s)* (**E**) mRNA in the liver (HFD^fre^, n = 8; HFD^fri^, n = 9); (**F**,**G**) expression of phospho-eukaryotic initiation factor 2α (p-eIF2α) (**F**) and activating transcription factor 4 (ATF4) (**G**) protein in the liver (n = 5); (**H**,**I**) relative expression of *Nicotinamide N-methyl transferase* (*Nnmt)* mRNA (**H**) and NNMT protein (**I**) in the liver (HFD^fre^, n = 8; HFD^fri^, n = 10). The adjusted *p*-values from multiple comparison tests are indicated as *, *p* < 0.05. The results of the Student *t*-test are provided in Supplementary Table S3.

We further explored the underlying mechanism of the liver damage by performing RNA-seq analysis on the liver (Supplementary Table S6). Principal component analysis (PCA) revealed remarkable differences in gene expression profiles (Figure 4A). In total, 445 genes with at least two-fold changes of expression (*p* < 0.05) were identified (Figure 4B). The top differentially expressed genes are presented as a heatmap (Supplementary Figure S3A). Gene set enrichment analysis (GSEA) showed that the upregulated genes induced by HFD^fri^ were enriched in the gene set related to UPR (Figure 4C). The expression of UPR-related genes was further confirmed by RT-qPCR (Supplementary Figure S3B).

We then elucidated the changes in signaling pathways related to UPR, including activating transcription factor 6 (ATF6) signaling, inositol-requiring enzyme 1 (IRE1) signaling, and PERK signaling. HFD^fri^ led to elevated levels of ATF6-related genes (Supplementary Figure S3C) and the upregulation of *Atf6* mRNA (Figure 4D). Similarly, dramatic elevation of the IRE1-related gene, *the spliced form of X-box binding protein 1 (Xbp1s)* (Figure 4E), and other relevant genes (Supplementary Figure S3D,E) were observed in the liver of mice fed HFD^fri^. The IRE1-related metabolite uridine diphospho-N-acetylglucosamine (UDP-GlcNAc) and its upstream metabolite glucosamine were also regulated in the liver of mice fed HFD^fri^ (Supplementary Figure S3F and Table S7). The increases of phospho-eukaryotic initiation factor 2α (p-eIF2α) and ATF4 suggested PERK signaling was also activated by HFD^fri^ (Figure 4F,G). Importantly, the expression of PERK-ATF4-regulated *Nnmt*NNMT mRNA (Figure 4H) and NNMT protein (Figure 4I) was also upregulated by HFD^fri^.

### 3.5. Hepatocyte-Specific Depletion of Nnmt Protected the Liver from MCD Diet-Induced Damages

**Figure 5:**
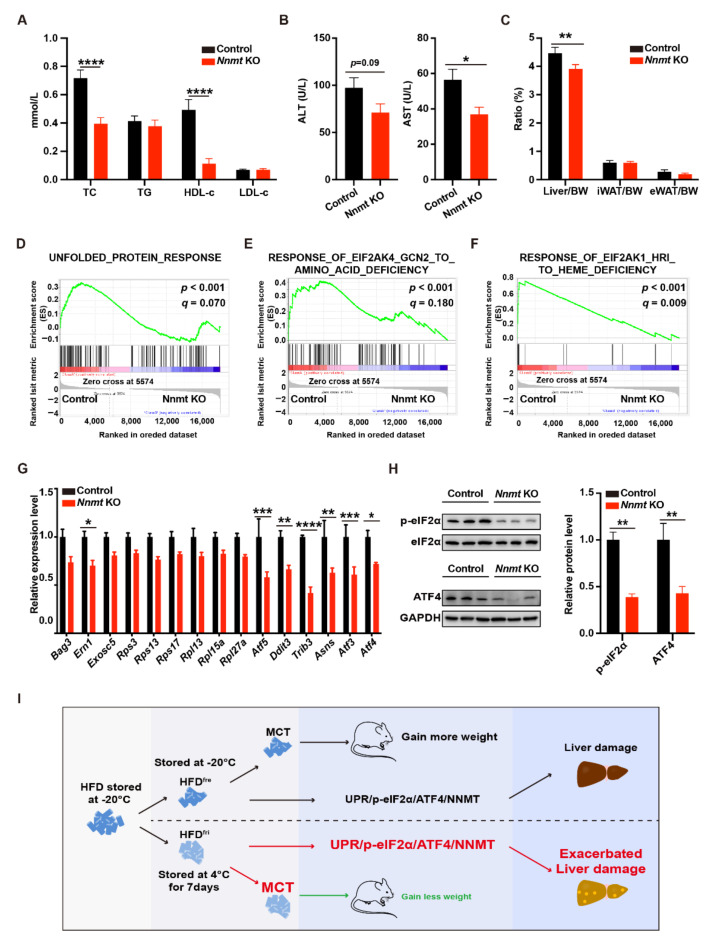
*Nicotinamide N-methyl transferase* knockout (NNMT KO) protected liver from methionine- and choline-deficient (MCD) diet-induced damages. (**A**,**B**) Analysis of the serum lipid profile ((**A**) *p* < 0.0001 from two-way ANOVA) and the serum concentration of alanine aminotransaminase (ALT) and aspartate aminotransferase (AST) (**B**) (control, n = 6; *Nicotinamide N-methyl transferase* knockout (NNMT KO), n = 7); (**C**) the proportion of liver, inguinal white adipose tissue (iWAT), and epididymal white adipose tissue (eWAT) (*p* = 0.0327 from two-way ANOVA; control, n = 6; NNMT KO, n = 7); (**D**–**F**) gene set enrichment analysis (GSEA) results of RNA-seq data in the liver (n = 4 for each group); (**G**) the expression of integrated stress response (ISR)-related genes in the liver (*p* < 0.0001 from two-way ANOVA, control, n = 4; NNMT KO, n = 6); (**H**) the expression of phospho-eukaryotic initiation factor 2α (p-eIF2α) and activating transcription factor 4 (ATF4) protein in the liver (*p* = 0.0005 from two-way ANOVA) (n = 3 for each group). The control or NNMT KO male mice were fed by methionine- and choline-deficient (MCD) diets, side-by-side, for 8 weeks. (**I**) Schematic figure of the study. The adjusted *p*-values from multiple comparison tests are indicated as *, *p* < 0.05; **, *p* < 0.01; ***, *p* < 0.001; ****, *p* < 0.0001 from the Student *t*-test. The results of the Student *t*-test are provided in Supplementary Table S3.

The changes of UPR-NNMT signaling in the liver inspired us to study the role of NNMT in dietary factor-induced liver damage. To this end, we employed a hepatocyte-specific *Nnmt* KO mouse model, *Alb-Cre::Nnmt ^flox/flox^* (referred to as *Nnmt* KO mice in the following), and an MCD diet-induced liver damage model. The KO efficiency was validated by RT-qPCR (Supplementary Figure S4A). Dramatic but comparable decreases in bodyweight were observed in both NNMT KO mice and control mice (NNMT*^flox/flox^*) fed on an MCD diet (Supplementary Figure S4B). Interestingly, lower concentrations of TC, ALT, and AST were observed in the serum of NNMT KO mice (Figure 5A,B). In addition, a lower proportion of liver, but not white adipose tissue (WAT), was identified in NNMT KO mice (Figure 5C and Supplementary Figure S4C). These results indicated that the knockout of NNMT protected the mice from dietary-induced liver damage.

We then investigated the mechanism of the protection provided by the knockout of NNMT (Supplementary Table S8). PCA revealed dramatic differences in gene expression profiles (Supplementary Figure S4D). In total, 390 genes with at least 2-fold changes of expression (*p* < 0.05) were identified (Supplementary Figure S4E). The top differentially expressed genes are presented in a heatmap (Supplementary Figure S4F). Interestingly, GSEA identified NNMT KO downregulated genes related to multiple ISR-related biological processes, including UPR, amino acid starvation, and heme deficiency (Figure 5D–F). The expression of representative genes is shown in Figure 5G. In addition, we observed decreases in phosphorylated eIF2α and ATF4 proteins in the NNMT KO mice (Figure 5H). These data revealed NNMT, as a downstream factor of UPR, regulated ISR in the context of liver damage. A scheme for current research is presented in Figure 5I.

## 4. Discussion

The diet is obviously an important factor affecting the key features of the DIO model. In the current study, we identified an unexpected body weight difference correlated with the storage conditions of HFDs. An untargeted LC-MS lipidomics analysis showed the enrichment of MCT in the HFD^fri^, which contributed to the variance of lipid absorption efficiency and obesity-related phenotypic changes. The HFD^fri^-induced liver damage is related to the activation of UPR. The hepatocyte-specific depletion of the UPR-regulated NNMT gene protected the MCD diet-receiving mice from liver damage via inhibition of the ISR.

There has been no consistent requirement for storage conditions of HFDs in metabolism studies. We have broadly consulted the users of multiple animal facilities in the USA and China, and they generally report that the storage conditions of HFDs after the package was opened are random (Supplementary Table S9). A comprehensive and systemic study is needed to understand whether the storage conditions are responsible for the experimental variations among these facilities.

It has been proposed by several studies that rodents and even humans taking MCT-enriched food may gain less fat mass [23,24,25,26,27]. This proposition is consistent with what we observed in the current study. We found the MCT-enriched oil had a lower absorption efficiency than the lipid mixture. This finding provides a mechanistic insight for the body weight differences induced by MCT.

We were surprised to find that the leaner mice fed by HFD^fri^ presented exacerbated liver damage. Notably, similar effects have been observed using an MCT-enriched diet [28]. Our study does not indicate that an MCT-enriched diet is not suitable for clinical application, as we were using mouse models. However, our results suggest clinicians need to design therapies for their patients carefully and consider the potential for liver damage.

Previously, we reported that the expression of hepatic NNMT was down-regulated by ketogenic diet feeding and upregulated by calorie restriction compared with chow-fed mice [7]. However, it remains unclear how the nutrients in the different diets affect the expression of hepatic NNMT. Here, we showed that mice fed with HFD^fri^ and HFD^fre^ had different levels of liver expression of NNMT. The different lipid composition of HFD^fri^ and HFD^fre^ regulates the NNMT expression, most likely through altered UPR status. The precise lipid components responsible for NNMT expression could be intriguing for further investigation.

The advanced liver damage and simultaneous elevation of UPR-NNMT signaling induced by HFD^fri^ inspired us to test the specific role of NNMT for diet-induced liver damage. It has been reported that whole-body depletion of NNMT leads to profound metabolic changes in mice [29], but the functions of NNMT in different tissues are variable [30].

With a hepatocyte-specific NNMT KO model, we identified the protective effects of NNMT depletion in MCD diet-induced liver damage. This observation is consistent with the protective effects of NNMT inhibition in carbon tetrachloride- or alcohol/palmitate-induced liver damage, suggesting the pivotal role of NNMT for liver diseases. Interestingly, NNMT depletion induced the downregulation of ISR in the liver. It is possible that there is a positive feedback loop connecting NNMT and ISR, whose disruption protects the liver from damages induced by various factors. The mechanism of how NNMT regulates ISR in the liver is interesting and needs to be studied further.

Our study is certainly limited by the fact that we used mice as model organisms. The HFD is also specially designed for the establishment of the DIO mouse model. It is not suitable to translate the findings directly into the real world. The role of NNMT in dietary factor-induced liver damage can be further confirmed by the comparisons between NNMT KO mice fed by HFD^fre^ and HFD^fri^, which can be performed in the future.

## 5. Conclusions

Our study provides evidence that HFD storage conditions can have a significant impact on both body weight changes and liver damage in the DIO model. NNMT may play important roles in dietary factor-induced liver damage.

## Data Availability

The raw data of this study was deposited to the Gene Expression Omnibus database at GSE203040.

## Supplementary Materials

The following supporting information can be downloaded at: https://www.mdpi.com/article/10.3390/nu14112222/s1, Figure S1: Physiological changes of mice fed by high fat diet stored in different conditions; Figure S2: The lipid composition in high fat diet stored in the fridge; Figure S3: Transcriptional and metabolic changes in the liver; Figure S4: The effects of nicotinamide N-methyl transferase KO in the liver; Table S1: Antibodies used in this study; Table S2: RT-PCR primers used in this study; Table S3: Student *t*-test and Two way ANOVA analysis of several figures; Table S4: Lipidomics of HFD^fre^ and HFD^fri^; Table S5: Lipidomics of serum after oral gavage of sunflower oil for 0 h or 2 h; Table S6: Genes transcripts per million (TPM) expression of mice liver fed by HFD^fre^ and HFD^fri^; Table S7: Genes transcripts per million (TPM) expression of Control and *Nnmt* Liver specific KO mice liver; Table S8: Metabolomics of mice liver fed by HFD^fre^ and HFD^fri^; Table S9: Storage conditions in different facility.
