# Supplementary material for: The Storage Conditions of High-Fat Diet Are the Key Factors for Diet-Induced Obesity and Liver Damage
Source: Nutrients. 2022 May 26;14(11):2222. doi: 10.3390/nu14112222 (PMC9182774; doi:10.3390/nu14112222)
Supplement: Supplementary file 1 [file nutrients-14-02222-s001.zip › nutrients-1722747-supplementary.pdf]

Supplementary information: The storage conditions of high fat diet are the key factors for diet induced obesity and liver damage

Chuanyou Yi[1,*], Dandan Li[1,*], Xiao Guo[1,*], Jinhui Wang[1], Cenxi Liu[1], Guangxing Lu[1], Yan Sun[2], He Huang [1,#], Shangyu Hong [1,#], Jin Li [1,#]

[1] State Key Laboratory of Genetic Engineering, School of Life Sciences, Human Phenome Institute, and Institute of metabolism and integrative biology, Fudan University, Shanghai, 200438, China

[2] Masonic medical research institute, 2150 Bleecker St, Utica, NY 13501, USA

[*] Equal contribution; [#] Correspondence: hehuang@ fudan.edu.cn (H.H.), shangyuhong@ fudan.edu.cn (S.H.) or li_jin_lifescience@fudan.edu.cn (J.L.)

Key word: high fat diet; storage condition; obesity; liver damage; medium chain triglyceride; unfolded protein response; NNMT.

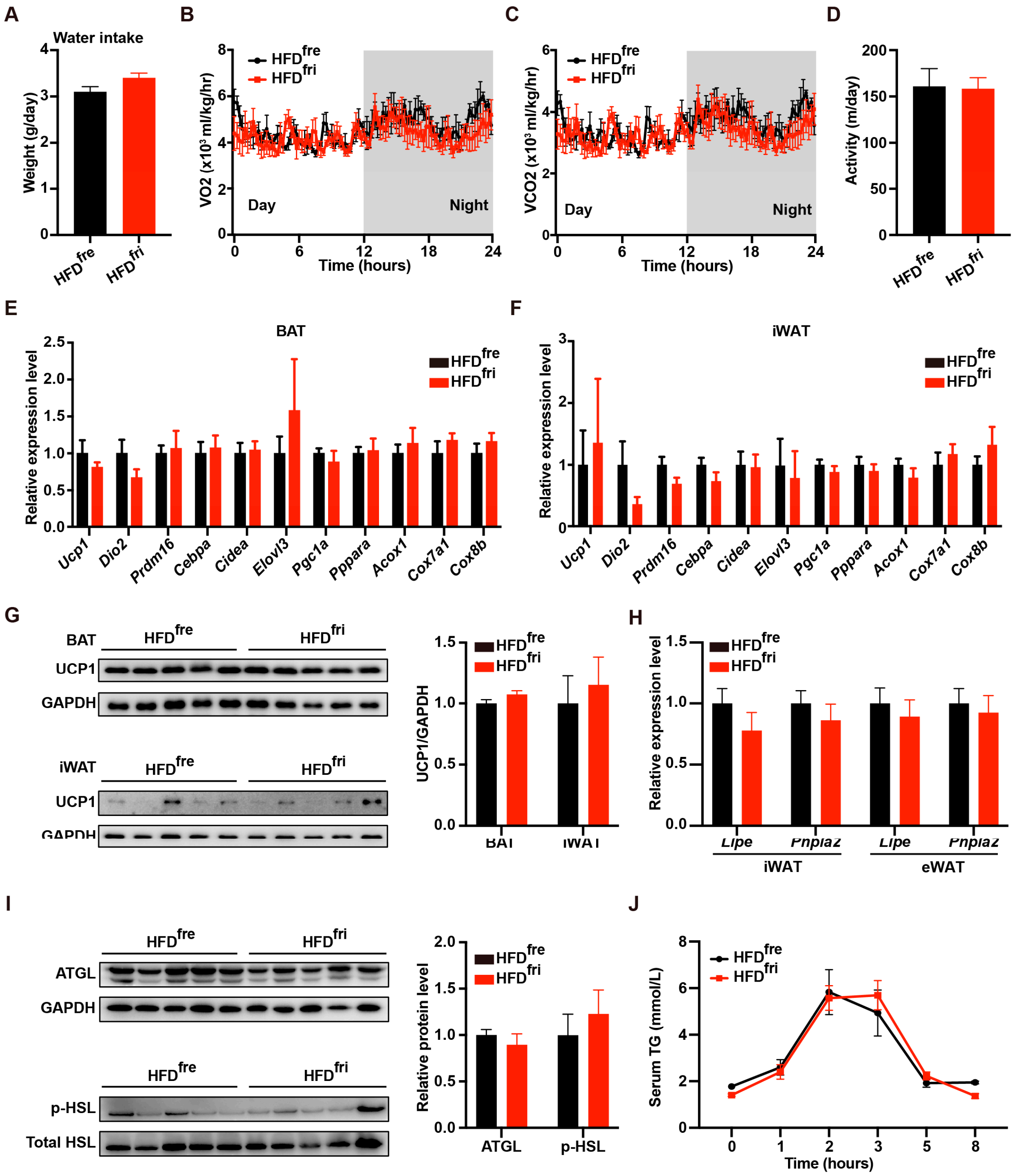

Supplementary Figure S1. Physiological changes of mice fed by high fat diet (HFD) stored in different conditions.

A-D. *Analysis of metabolic features of mice fed by high fat diet (HFD) stored in the freezer (fre) (HFD^{fre}) or HFD stored in the fridge (fri) (HFD^{fri}). The features include (A) water intake (n=9 for each group); (**B**) O_2 consumption rate at each time point (n=4 for each group); (**C**) CO_2 production rate at each time point (n=4 for each group) and (**D**) physical activity (n=4 for each group);* ***E.*** *The expression of thermogenic genes in brown adipose tissue (BAT) (n=10 for each group);* ***F.*** *The expression of*

thermogenic genes in inguinal white adipose tissue (iWAT) (n=10 for each group); ***G.*** *The expression of uncoupling protein (UCP1) protein in BAT and iWAT (n=5 for each group);* ***H.*** *The expression of lipolytic genes in iWAT and epidydimal white adipose tissue (eWAT) (n=10 for each group);* ***I.*** *The expression of lipolytic proteins in iWAT and eWAT (n=5 for each group);* ***J.*** *The oral lipid tolerance test (OLTT) results of mice fed by HFD stored in different conditions (p=0.8452 from two-way ANOVA, n=10 for each group). Eight-week-old male mice were fed by* HFD^{fre} *and* HFD^{fri} *for 7 weeks. The adjusted p-value from multiple comparison test and the p-value from student t-test were provided in the* ***Supplementary Table S3****.*

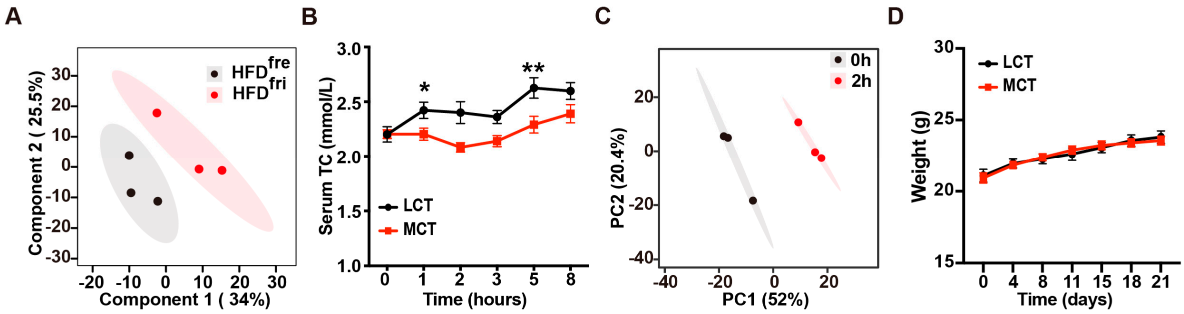

Supplementary Figure S2. The lipid composition in high fat diet (HFD) stored in the fridge (fri) (HFD^{fri}).

A. *The partial least squares discrimination analysis (PLS-DA) of lipidomics analysis for HFD (n=3 for each group);* ***B.*** *The serum total cholesterol (TC) concentration in oral lipid tolerance test (OLTT) in eight-week-old male mice fed by chow diet ($p<0.0001$ from two-way ANOVA, n=10 for each group);* ***C.*** *The principal component analysis (PCA) of serum lipidomics in the initial and two-hour time point of OLTT (n=3 for each group);* ***D.*** *The body weight of eight-week-old male mice fed by chow diet and medium chain triglyceramide (MCT) enriched oil or sunflower oil for three weeks ($p=0.9209$ from two-way ANOVA and n=10 for each group). The adjusted p-value from multiple comparison test were indicated as *, $p<0.05$; **, $p<0.01$. The results of student t-test were provided in the* ***Supplementary Table S3****.*

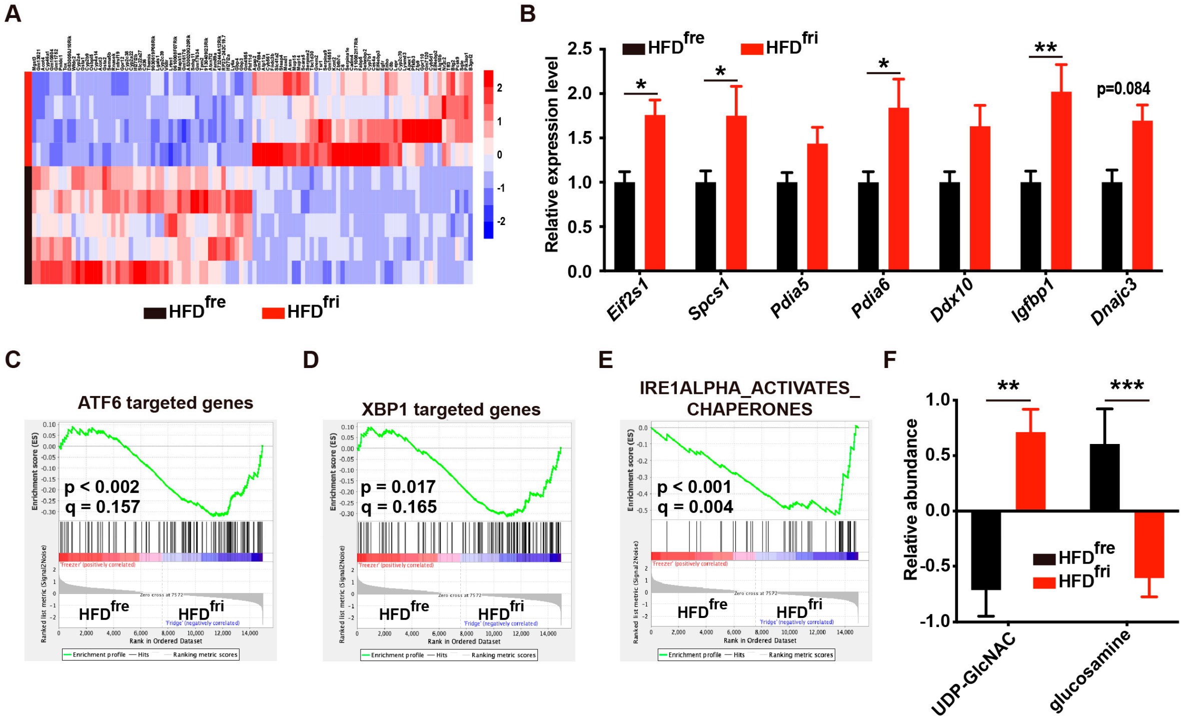

Supplementary Figure S3. Transcriptional and metabolic changes in the liver. ***A.*** *The heatmap of the RNA-seq data (high fat diet (HFD) stored in the freezer (fre) (HFD^{fre}), n=5; HFD stored in the fridge (fri) (HFD^{fri}), n=4);* ***B-F.*** *Transcriptional changes and metabolic changes of liver from mice fed by HFD. These include (**B**) RT-qPCR results of genes related to unfolded protein response (UPR) ($p<0.0001$ from two-way ANOVA; HFD^{fre}, n=8; HFD^{fri}, n=7); (**C**) Gene Set Enrichment Analysis (GSEA) result for activating transcription factor 6 (ATF6) associated gene set (HFD^{fre}, n=5; HFD^{fri}, n=4); (**D**) X-box binding protein 1 (XBP1) associated gene set set (HFD^{fre}, n=5; HFD^{fri}, n=4); (**E**) Endoplasmic reticulum (ER) related chaperon associated gene set set (HFD^{fre}, n=5; HFD^{fri}, n=4) and (**F**) Relative abundance of metabolites in the liver set (n=10 for each group). Eight-week-old male mice were fed by HFD^{fre} and HFD^{fri} for 7 weeks. The adjusted p-value from multiple comparison test were indicated as *, $p<0.05$; **, $p<0.01$. The results of student t-test were provided in the* ***Supplementary Table S3****.*

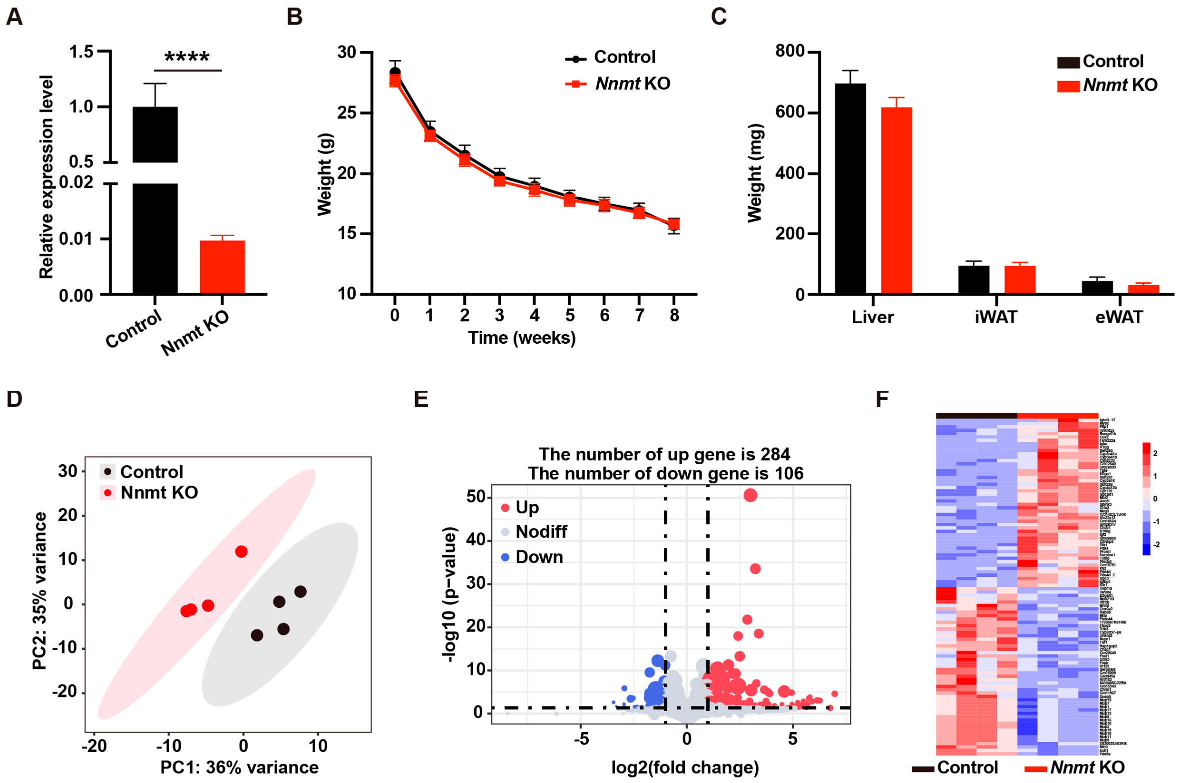

Supplementary Figure S4. The effects of nicotinamide N-methyl transferase knockout (Nnmt KO) in the liver.

A. *Expression of nicotinamide N-methyl transferase (Nnmt) gene by RT-qPCR (n=3 for each group);* ***B.*** *Body weight (p=0.2553 from two-way ANOVA; Control, n=6; nicotinamide N-methyl transferase knockout (Nnmt KO), n=7);* ***C.*** *Absolute weight of liver, inguinal white adipose tissue (iWAT) and epididymal white adipose tissue (eWAT) (p=0.1199 from two-way ANOVA; Control, n=6; Nnmt KO, n=7);* ***D.*** *Principal Component Analysis (PCA) (n=4 for each group);* ***E.*** *Volcano plot (n=4 for each group);* ***F.*** *Heat map of top differentially expressed genes in the liver of male control or Nnmt KO mice fed by methionine and choline deficient (MCD) diet side by side for eight weeks (n=4 for each group). ****, p<0.0001 from student t-test. The adjusted p-value from multiple comparison test and the results of student t-test were provided in the* ***Supplementary Table S3****.*
